# Supplementary material for: A Branching Process to Characterize the Dynamics of Stem Cell Differentiation
Source: Sci Rep. 2015 Aug 19;5:13265. doi: 10.1038/srep13265 (PMC4541069; doi:10.1038/srep13265)
Supplement: Supplementary Information [file srep13265-s1.pdf]

A Branching Process to Characterize the Dynamics of Stem Cell Differentiation

David G. Míguez*

July 1, 2015

Depto. de Física de la Materia Condensada, Instituto Nicolás Cabrera and IFIMAC, Universidad Autónoma de Madrid, Campus de Cantoblanco, 28046 Madrid, Spain

* Corresponding Author: David G. Míguez: david.gomez.miguez@uam.es

Keywords: Stem Cell, Differentiation, Neurogenesis, Development, Branching processes, Mathematical Models, Systems Biology

Running Title: A Branching Process to study stem cell differentiation

Supplementary Data

Derivation of the equations

Eqs.1-3 in the main text can be derived following an iterative process, initially assuming discrete time points in multiples of the average cell cycle $n = \triangle t/T$, (n=1,2,3...) to then generalize to the continuum limit. We start from a initial population of progenitors P_0 and differentiated D_0 cells. In principle, we assume that a fraction γ of the progenitor population is cycling with average cell cycle T (i.e, the remaining progenitor population $P_0(1-\gamma)$ is in quiescence state). Therefore, taking into account that each pp division generates two progenitor cells, each pd division generates one progenitor and one differentiated cell, dd divisions generate two differentiated cells, while $\emptyset$ generates one dead cell, for n=1 we can write the number of progenitor, differentiated and dead cells as:

$$\begin{aligned} P_1 &= P_0\gamma(2pp + pd) + P_0(1-\gamma) && (1)\\ D_1 &= D_0 + P_0\gamma(2dd + pd) && (2)\\ \Psi_1 &= \emptyset P_0 && (3) \end{aligned}$$

where using the condition $pp + pd + dd + \emptyset = 1$,

$$\begin{aligned} P_1 &= P_0(1 + \gamma(pp - dd - \emptyset)) && (4)\\ D_1 &= D_0 + P_0\gamma(1 + dd - pp - \emptyset) && (5)\\ \Psi_1 &= \emptyset P_0 && (6) \end{aligned}$$

For n=2,

$$\begin{aligned} P_2 &= P_1(1 + \gamma(pp - dd - \emptyset)) && (7)\\ D_2 &= D_1 + P_1\gamma(1 + dd - pp - \emptyset) && (8)\\ \Psi_2 &= \Psi_1 + \emptyset P_1 && (9) \end{aligned}$$

applying Eqs. 4-6, we obtain

$$\begin{aligned} P_2 &= P_0(1 + \gamma(pp - dd - \emptyset))(1 + \gamma(pp - dd - \emptyset)) = P_0(1 + \gamma(pp - dd - \emptyset))^2 && (10)\\ D_2 &= D_0 + P_0\gamma(1 + dd - pp - \emptyset) + P_0(1 + \gamma(pp - dd - \emptyset))\gamma(1 + dd - pp - \emptyset) && (11)\\ \Psi_2 &= \emptyset P_0 + \emptyset P_0(1 + \gamma(pp - dd - \emptyset)) = \emptyset P_0(1 + (1 + \gamma(pp - dd - \emptyset))) && (12) \end{aligned}$$

and rearranging terms in eq. 11

$$\begin{aligned} D_2 &= D_0 + P_0\gamma(1 + dd - pp - \emptyset)(1 + (1 + \gamma(pp - dd - \emptyset))) && (13)\\ & && (14) \end{aligned}$$

Subsequently, for n=3

$$\begin{aligned} P_3 &= P_2(1 + \gamma(pp - dd - \emptyset)) && (15)\\ D_3 &= D_2 + P_2\gamma(1 + dd - pp - \emptyset) && (16)\\ \Psi_3 &= \Psi_2 + \emptyset P_2 && (17) \end{aligned}$$

applying eqs. 10-12, we obtain

$$\begin{aligned} P_3 &= P_0(1 + \gamma(pp - dd - \emptyset))^2(1 + \gamma(pp - dd - \emptyset)) = P_0(1 + \gamma(pp - dd - \emptyset))^3 && (18)\\ D_3 &= D_0 + P_0\gamma(1 + dd - pp - \emptyset)(1 + (1 + \gamma(pp - dd - \emptyset)) + (1 + \gamma(pp - dd - \emptyset))^2) && (19)\\ \Psi_3 &= \emptyset P_0(1 + (1 + \gamma(pp - dd - \emptyset)) + (1 + \gamma(pp - dd - \emptyset))^2) && (20) \end{aligned}$$

therefore, for n steps, we obtain,

$$\begin{aligned} P_n &= P_0(1 + \gamma(pp - dd - \emptyset))^n && (21)\\ D_n &= D_0 + P_0\gamma(1 + dd - pp - \emptyset) \times\\ &\quad \times(1 + (1 + \gamma(pp - dd - \emptyset)) + (1 + \gamma(pp - dd - \emptyset))^2 + ... + \gamma(pp - dd - \emptyset))^{n-1}) && (22)\\ \Psi_n &= \emptyset P_0(1 + (1 + \gamma(pp - dd - \emptyset)) + (1 + \gamma(pp - dd - \emptyset))^2 + ... + \gamma(pp - dd - \emptyset))^{n-1}) && (23) \end{aligned}$$

where the second term in eqs. 22-23 can be written as

$$1+(1+\gamma(pp-dd-\emptyset))+(1+\gamma(pp-dd-\emptyset))^2+...+\gamma(pp-dd-\emptyset))^{n-1}=\sum_{i=0}^{n-1}(1+\gamma(pp-dd-\emptyset))^i \quad (24)$$

which renaming $r=1+\gamma(pp-dd-\emptyset)$ is equivalent to

$$\sum_{i=0}^{n-1} r^i = \frac{1-r^n}{1-r} \quad (25)$$

therefore, eqs. 22-23 can be written as

$$D_n = D_0 + P_0\gamma(1+dd-pp-\emptyset)\frac{1-(1+\gamma(pp-dd-\emptyset))^n}{1-(1+\gamma(pp-dd-\emptyset))} \quad (26)$$

$$\Psi_n = \emptyset P_0\frac{1-(1+\gamma(pp-dd-\emptyset))^n}{1-(1+\gamma(pp-dd-\emptyset))} \quad (27)$$

which, after simplifying terms, can be rewritten as

$$D_n = D_0 + P_0(1-(1+\gamma(pp-dd-\emptyset))^n)\frac{1+dd-pp-\emptyset}{dd+\emptyset-pp} \quad (28)$$

$$\Psi_n = P_0((1+\gamma(pp-dd-\emptyset))^n-1)\frac{\emptyset}{\gamma(pp-dd-\emptyset)} \quad (29)$$

and taking into account eq. 21, we obtain the final form of eqs. 2-3 in the main text for discrete time steps:

$$D_n = D_0 + (P_0-P_n)\frac{1+dd-pp-\emptyset}{dd+\emptyset-pp} = D_0 + (P_n-P_0)\frac{1+dd-pp-\emptyset}{pp-dd-\emptyset} \quad (30)$$

$$\Psi_n = \frac{\emptyset(P_n-P_0)}{\gamma(pp-dd-\emptyset)} \quad (31)$$

Interestingly, both equations depend on the iteration step n only via the number of progenitors at a given time in the system P_n. For simplicity, the system of equations has been derived for a situation of discrete $n=\triangle t/T$, (n=1,2,3...), i.e., with the time step equal to the average cell cycle $\triangle t = T$. If we instead consider the time step as half of the cell cycle ($\triangle t = T/2$), then $n = 2\triangle t/T$, (n=1,2,3...), and eq. 4 is now:

$$P_1 = P_0(1+\gamma(pp-dd-\emptyset))^{\frac{1}{2}} \quad (32)$$

and following identical iteration steps we arrive at

$$P_n = P_0(1+\gamma(pp-dd-\emptyset))^{\frac{n}{2}} \quad (33)$$

while eqs. 30-32 for differentiated and dead cells remain the same, since they do not depend explicitly on the iteration step. This way, for $\triangle t = 1$, and following the same process we obtain

$$P_n = P_0(1+\gamma(pp-dd-\emptyset))^{\frac{n}{T}} \quad (34)$$

which can be generalize for the continuum limit $n=t$,

$$P_t = P_0(1+\gamma(pp-dd-\emptyset))^{\frac{t}{T}} \quad (35)$$

with 30-32 only depend on time via the number of progenitors P_t. This means that, for fixed values of division and apoptosis rates, the number of differentiated and dead cells at a given time t can be calculated based on the number of progenitors that remain in the system at this time.

$$D_t = D_0 + (P_t-P_0)\frac{1+dd-pp-\emptyset}{pp-dd-\emptyset} \quad (36)$$

$$\Psi_t = \frac{\emptyset(P_t-P_0)}{\gamma(pp-dd-\emptyset)} \quad (37)$$

that, if we rewrite $\Delta P = P_t - P_0$, we obtain the expression of Eqs. 2-3 in the main text.

$$D_t = D_0 + \Delta P \frac{1 + dd - pp - \emptyset}{pp - dd - \emptyset} \tag{38}$$

$$\Psi_t = \Delta P \frac{\emptyset}{\gamma(pp - dd - \emptyset)} \tag{39}$$

Validation and calculation of equation for total number of cells

After each cell cycle, any given pool of proliferating and differentiating cycling progenitors produces a total amount of cells that is twice the amount of dividing cells (i.e, $P(t+T) + D(t+T) = 2P_t$ in conditions of no apoptosis, independently of the time of division taking place. To derive an expression for the total number of cells A at any given time in the system, we add eqs. 35-37:

$$A(t) = P_0(1 + \gamma(pp - dd - \emptyset))^{\frac{t}{T}} + D_0 + (P_t - P_0)\frac{1 + dd - pp - \emptyset}{pp - dd - \emptyset} + \frac{\emptyset(P_t - P_0)}{\gamma(pp - dd - \emptyset)} \tag{40}$$

and rearranging terms

$$A(t) = D_0 + P_0((1 + \gamma(pp - dd - \emptyset))^{\frac{t}{T}} + ((1 + \gamma(pp - dd - \emptyset))^{\frac{t}{T}} - 1)\frac{\emptyset + \gamma(1 + dd - pp - \emptyset)}{\gamma(pp - dd - \emptyset)}) \tag{41}$$

$$A(t) = D_0 + P_0((1 + \gamma(pp - dd - \emptyset))^{\frac{t}{T}} + ((1 + \gamma(pp - dd - \emptyset))^{\frac{t}{T}} - 1)\frac{\emptyset + \gamma(1 + dd - pp - \emptyset)}{\gamma(pp - dd - \emptyset)}) \tag{42}$$

which at $t = T$ (i.e, one cell cycle length) and in conditions of no apoptosis ($\emptyset = 0$), eq. 42 becomes:

$$A(t) = D_0 + P_0(1 + \gamma(pp - dd) + \gamma(1 + dd - pp)) = D_0 + P_0(1 + \gamma) \tag{43}$$

which if all progenitors are cycling ($\gamma = 1$), we obtain $A(t) = D_0 + 2P_0$, as expected.

Homeostasis condition

In conditions of homeostasis in the progenitor population (i.e., $pp - dd - \emptyset = 0$), eqs. 2-3 in the main text are indeterminate form of the type 0/0. Therefore, to obtain the value of differentiated and dead cells in the case of maintenance of the progenitor population, we apply L'Hopital's rule ($\lim_{x \to c} \frac{f(x)}{g(x)} = \lim_{x \to c} \frac{f'(x)}{g'(x)}$). If we identify $pp - dd - \emptyset = x$, eqs. 2-3 in the main text can be written as:

$$D_t = D_0 + P_0((1 + \gamma x)^{\frac{t}{T}} - 1)\frac{1 - x - 2\emptyset}{x} \tag{44}$$

$$\Psi_t = P_0((1 + \gamma x)^{\frac{t}{T}} - 1)\frac{\emptyset}{\gamma x} \tag{45}$$

after derivation of numerator and denominator we obtain.

$$\lim_{x \to 0}(D_t - D(0)) = \lim_{x \to 0} \frac{P_0((1 + \gamma x)^{\frac{t}{T}} - 1)(1 - x - 2\emptyset)}{x} \stackrel{\mathrm{H}}{=} \frac{P(0)\gamma t(1 - 2\emptyset)}{T} \tag{46}$$

$$\lim_{x \to 0} \Psi_t = \lim_{x \to 0} \frac{\emptyset P_0((1 + \gamma x)^{\frac{t}{T}} - 1)}{\gamma x} \stackrel{\mathrm{H}}{=} \emptyset P(0)\frac{t}{T} \tag{47}$$

which in conditions of constant cell cycle T and apoptosis rate $\emptyset$, corresponds to linear growth of the population of differentiated cells D_t and amount of dead cells Ψ_t overtime.

Calculation of number of total cells in growing tissues based on data from sections

Equations presented here are valid for systems where the total number of cells can be quantified at different times. In the context of the developing spinal cord, quantification of the total amount of cells generated

is not possible using conventional wide field or confocal microscopy techniques. Instead, scientist focused on transversal sections of the spinal cord to study the dynamics of proliferation and differentiation (1; 2). In its present form, the equations presented here cannot be used to measure cell cycle length based on the cells in a transversal section, since a number of cells generated in a given section will contribute to the anteroposterior growth of the spinal cord. Recently, the anterioposterior rate of grwoth of the chick spinal cord has been estimated by photo-conversion of fluorescent proteins and measuring the change in width of of a given section of the spinal cord after a given time (3). This data shows that after 15 hours, a photo converted stripe grows from 140 μm to 173 μm, which represents a 15% anteroposterior growth in 15 hours (i.e., 1% growth per hour). If we assume, as a first approximation, that this axial growth is constant, we can estimate that, on average, 1% of the cells generated in a given section per hour leave the section to contribute to the antero-posterior growth of the signal cord. Assuming this as a constant rate, we can estimate the total number of cells generated in a section based on the quantification in Fig. 2B (see Supplementary Fig. 3C).

To calculate the corrected average cell cycle and mode of division, we will generate a set of equations to account for the rate of cells being lost from a given section per unit time α, such as, for n=1:

$$P_1^* - P_0^* = (1+\alpha)(P_1 - P_0) \tag{48}$$
$$D_1^* - D_0^* = (1+\alpha)(D_1 - D_0) \tag{49}$$
$$\Psi_1^* - \Psi_0^* = (1+\alpha)(\Psi_1 - \Psi_0) \tag{50}$$

where P_i^*, D_i^* and Ψ_i^* correspond to the absolute number of progenitor, differentiated and dead cells that have been generated in a given section, and P_i, D_i and Ψ_i corresponds to the cells that remain in the section. Therefore, following the same rationale as in eqs. 1-3, we can write:

$$P_1^* = P_0^*\gamma(2pp+pd) + P_0^*(1-\gamma) + \alpha(P_1 - P_0^*) \tag{51}$$
$$D_1^* = D_0^* + P_0^*\gamma(2dd+pd) + \alpha P_0^*\gamma(2dd+pd) \tag{52}$$
$$\Psi_1^* = \emptyset P_0^* + \alpha\emptyset P_0^* \tag{53}$$

Substituting $P_1 = P_0^*(1+\gamma(pp-dd-\emptyset))$, and using the condition $pp+pd+dd+\emptyset = 1$, we can rearrange terms and obtain:

$$P_1^* = P_0^*(1+\gamma(pp-dd-\emptyset) + \alpha(1+\gamma(pp-dd-\emptyset)-1)) = P_0^*(1+\gamma(pp-dd-\emptyset)(\alpha+1)) \tag{54}$$
$$D_1^* = D_0^* + P_0^*\gamma(1+dd-pp-\emptyset)(1+\alpha) \tag{55}$$
$$\Psi_1^* = \emptyset P_0^*(1+\alpha) \tag{56}$$

In the same way, for n=2:

$$P_2^* = P_1^*\gamma(2pp+pd) + P_1^*(1-\gamma) + \alpha(P_2 - P_1^*) \tag{57}$$
$$D_2^* = D_1^* + P_1^*\gamma(2dd+pd) + \alpha P_1^*\gamma(2dd+pd) \tag{58}$$
$$\Psi_2^* = \Psi_1^* + \emptyset P_1^*(1+\alpha) \tag{59}$$

where substituting eq. 55 and eq. 56 into eq. 58 and eq. 59, respectively, we obtain

$$P_2^* = P_1^*(1+\gamma(pp-dd-\emptyset)(\alpha+1)) = P_0^*(1+\gamma(pp-dd-\emptyset)(\alpha+1))^2 \tag{60}$$
$$D_2^* = D_0^* + P_0^*\gamma(1+dd-pp-\emptyset)(1+\alpha) + P_1^*\gamma(1+dd-pp-\emptyset)(1+\alpha) \tag{61}$$
$$\Psi_2^* = \emptyset P_0^*(1+\alpha) + \emptyset P_0^*(1+\gamma(pp-dd-\emptyset)(\alpha+1))(1+\alpha) \tag{62}$$

and and rearranging terms in 61-62:

$$D_2^* = D_0^* + P_0^*\gamma(1+dd-pp-\emptyset)(1+\alpha)(1+(1+\gamma(pp-dd-\emptyset)(\alpha+1))) \tag{63}$$
$$\Psi_2^* = \emptyset P_0^*(1+\alpha)(1+(1+\gamma(pp-dd-\emptyset)(\alpha+1)) \tag{64}$$

Therefore, for an arbitrary n multiples of the cell cycle, we obtain:

$$P_n^* = P_0^*(1+\gamma(pp-dd-\emptyset)(\alpha+1))^n \quad (65)$$

$$D_n^* = D_0^* + P_0^*\gamma(1+dd-pp-\emptyset)(1+\alpha)\times \times(1+(1+\gamma(pp-dd-\emptyset)(\alpha+1))+...+\gamma(pp-dd-\emptyset)(\alpha+1))^{n-1}) \quad (66)$$

$$\Psi_n^* = \emptyset P_0^*(1+\alpha)(1+(1+\gamma(pp-dd-\emptyset)(\alpha+1))+...+(1+\gamma(pp-dd-\emptyset)(\alpha+1))^{n-1} \quad (67)$$

where the second term of eqs. 66-67 can be written and solved in the same form as eq. 26. Therefore, eqs. 66-67 can be written as

$$D_n^* = D_0^* + P_0^*\gamma(1+dd-pp-\emptyset)(1+\alpha)\frac{1-(1+\gamma(pp-dd-\emptyset)(1+\alpha))^n}{1-(1+\gamma(pp-dd-\emptyset)(1+\alpha))} \quad (68)$$

$$\Psi_n^* = \emptyset P_0^*(1+\alpha)\frac{1-(1+\gamma(pp-dd-\emptyset)(1+\alpha))^n}{1-(1+\gamma(pp-dd-\emptyset)(1+\alpha))} \quad (69)$$

after applying eq. 65 and simplifying:

$$D_n^* = D_0^* + (P_n^*-P_0^*)\frac{\gamma(1+dd-pp-\emptyset)(1+\alpha)}{1-(1+\gamma(pp-dd-\emptyset)(1+\alpha))} = D_0^* + (P_n^*-P_0^*)\frac{1+dd-pp-\emptyset}{pp-dd-\emptyset} \quad (70)$$

$$\Psi_n^* = (P_n^*-P_0^*)(1+\alpha)\frac{\emptyset(1+\alpha)}{1-(1+\gamma(pp-dd-\emptyset)(1+\alpha))} = \frac{\emptyset(P_n^*-P_0^*)}{\gamma(pp-dd-\emptyset)} \quad (71)$$

that do not depend explicitly on the amount of generated cells that leave the system α, only implicitly through the term P_n^*. Finally, these set of equations can be also generalized for any given time, taking the form:

$$P_t^* = P_0^*(1+\gamma(pp-dd-\emptyset)(\alpha+1))^{t/T} \quad (72)$$

$$D_t^* = D_0^* + (P_t^*-P_0^*)\frac{1+dd-pp-\emptyset}{pp-dd-\emptyset} \quad (73)$$

$$\Psi_t^* = \frac{\emptyset(P_t^*-P_0^*)}{\gamma(pp-dd-\emptyset)} \quad (74)$$

and substituting $\Delta P^* = P_t^* - P_0^*$,

$$P_t^* = P_0^*(1+\gamma(pp-dd-\emptyset)(\alpha+1))^{t/T} \quad (75)$$

$$D_t^* = D_0^* + \Delta P^*\frac{1+dd-pp-\emptyset}{pp-dd-\emptyset} \quad (76)$$

$$\Psi_t^* = \Delta P^*\frac{\emptyset}{\gamma(pp-dd-\emptyset)} \quad (77)$$

Calculation of mode and type of division in growing tissues based on data from sections

To determine the mode of division and cell cycle length based on data from transversal sections in tissues that growth in the axial direction we will take advantage of the previous equations for progenitors and differentiated cells. Rearranging eqs. 72-73:

$$pp-dd-\emptyset = \frac{\Delta P^*(1-2\emptyset)}{\Delta D^* + \Delta P^*} \quad (78)$$

$$T = t\frac{log(1+\gamma(pp-dd-\emptyset)(\alpha+1))}{log\frac{P_t^*}{P_0^*}} = t\frac{log(1+\gamma\frac{\Delta P^*(1-2\emptyset)(\alpha+1)}{\Delta D^*+\Delta P^*})}{log\frac{P_t^*}{P_0^*}} \quad (79)$$

where $\Delta P^* = P_t^* - P_0^*$ and $\Delta D^* = D_t^* - D_0^*$, as defined in eqs. 48-50. Since α has been defined as the rate of generated cells leaving the section, such as ΔP^*=$(1+\alpha)\Delta P$ and ΔD^*=$(1+\alpha)\Delta D$, we can rewrite eqs. 78 as

$$pp - dd - \emptyset \quad = \quad \frac{\Delta P(1+\alpha)(1-2\emptyset)}{(1+\alpha)(\Delta P + \Delta D)} = \frac{\Delta P(1-2\emptyset)}{\Delta P + \Delta D} \tag{80}$$

which is equivalent to eq. 4 in the main text, and shows that the calculation of the mode of division based on data from sections of an axially growing tissue is equivalent as the value obtained taking into account the cells that leave the system. On the other hand, eq. 79 becomes:

$$T \quad = \quad t\frac{log(1+\gamma\frac{\Delta P(\alpha+1)(1-2\emptyset)}{\Delta P+\Delta D}}{log\frac{P_t^*}{P_0^*}} = t\frac{log(1+\gamma\frac{\Delta P^*(1-2\emptyset)}{\Delta P+\Delta D}}{log\frac{P_t^*}{P_0^*}} \tag{81}$$

which is not equivalent to eq. 6 in the main text, since both numerator and denominator still depend on the absolute number of cells P^* and P_0^*. This is teh equation used to compute the value of the absolute cell cycle to be compared with the experimental values in Fig. 2E.

Binomial distribution approach to calculate the rates of division

In a potential scenario where the fate of both daughter cells after a mitosis is considered as independent events, one can define a binomial experiment with two mutually exclusive outcomes, often referred to as "success" and "failure". Lets consider "success" the event of remaining a progenitor cell and "failure" will be the event of undergoing differentiation. Lets define the probability of success as q, and the probability of failure as $1-q$ (i.e., $1 \geqslant q \geqslant 0$). The "success probability" can be written in terms of the rates of division $pp - dd$, defined in the text as the rate of generation of new progenitor cells per division event (the rate of apoptosis in neglected in this derivation). Therefore, $q = (1 + pp - dd)/2$, in such a way that $q = 1$ when all division are pp and all the generated cells are progenitors, $q = 0$ when all division are dd and all generated cells are differentiation, and $q = 0.5$ when $pp - dd = 0$, i.e., half of the generated cells are progenitors while the other half are differentiated cells.

When considering the decision between proliferation and differentiation as purely random decision, it can be considered as a Bernoulli trial, with the random variable x as a function of the number of trials n and the "success" probability $q = \frac{1+pp-dd}{2}$:

$$x \sim B(n, \frac{1+pp-dd}{2}) \tag{82}$$

and a probability distribution defined as:

$$P(X = x) = \binom{n}{x}(\frac{1+pp-dd}{2})^x \cdot (1 - \frac{1+pp-dd}{2})^{n-x} \tag{83}$$

This way, in an experiment with two trials ($n = 2$), the probabilities of the two cells to become progenitors ($x = 2$) or differentiated cells ($x = 0$) are:

$$P(X = 2) = \binom{2}{2}(\frac{1+pp-dd}{2})^2 \cdot (1 - \frac{1+pp-dd}{2})^{2-2} = (\frac{1+pp-dd}{2})^2 \tag{84}$$

$$P(X = 0) = \binom{2}{0}(\frac{1+pp-dd}{2})^0 \cdot (1 - \frac{1+pp-dd}{2})^{2-0} = (\frac{1+dd-pp}{2})^2 \tag{85}$$

as expected. On the other hand, an experiment with two trials ($n = 2$) where just one of the cells becomes a progenitor (i.e., just one of the cell "succeeds", $x = 1$) has a probability of

$$P(X = 1) = \binom{2}{1}(\frac{1+pp-dd}{2})^1 \cdot (1 - \frac{1+pp-dd}{2})^{2-1} = 2(\frac{1+pp-dd}{2}) \cdot (1 - \frac{1+pp-dd}{2}) \tag{86}$$

that, after rearranging terms, becomes,

$$P(X=1) = (1+pp-dd)\cdot(\frac{1-pp+dd}{2}) = \frac{1}{2}(1-(pp-dd)^2) \tag{87}$$

This probability can be identified as the rate of pd divisions in systems where the decision between proliferation of differentiation of the two daughter cells of a division event is stochastic and independent of each other. Then, by substitution into eq. 4 in the main text for conditions of negligible apoptosis, we obtain:

$$pd = \frac{1}{2}(1-(\frac{\Delta P}{\Delta D+\Delta P})^2) = \frac{\Delta D(2\Delta P+\Delta D)}{2(\Delta P+\Delta D)^2} \tag{88}$$

To obtain the equation for dd, we rewrite eq. 4 in the main text for no apoptosis, taking into account that $pp+pd+dd=1$:

$$pp-dd = 1-pd-2dd = \frac{\Delta P}{\Delta D+\Delta P} \tag{89}$$

and rearranging terms after substituting eq. 88 we obtain:

$$2dd = 1-\frac{\Delta P}{\Delta D+\Delta P}-pd = \frac{\Delta D}{\Delta D+\Delta P}-pd = \frac{\Delta D}{\Delta D+\Delta P}(1-\frac{2\Delta P+\Delta D}{2(\Delta P+\Delta D)}) \tag{90}$$

that after rearranging terms becomes

$$dd = \frac{\Delta D}{2(\Delta D+\Delta P)}\frac{2\Delta P+2\Delta D-2\Delta P-\Delta D}{2(\Delta P+\Delta D)} = (\frac{\Delta D}{2(\Delta D+\Delta P)})^2 \tag{91}$$

Finally, the expression for pp can be obtained by substituting eq. 91 into eq. 4 in the main text:

$$pp = \frac{\Delta P}{\Delta D+\Delta P}+dd = \frac{\Delta P}{\Delta D+\Delta P}+\frac{\Delta D^2}{4(\Delta D+\Delta P)^2} \tag{92}$$

Again, by rearranging terms we obtain:

$$pp = \frac{4\Delta P(\Delta D+\Delta P)+\Delta D^2}{4(\Delta D+\Delta P)^2} = \frac{\Delta P\cdot\Delta D+\Delta P^2)+\frac{1}{4}\Delta D^2}{(\Delta D+\Delta P)^2} = (\frac{\Delta P+\frac{1}{2}\Delta D}{\Delta D+\Delta P})^2 \tag{93}$$

Validation of rates of division derived using binomial approach

In this section, we will show that the expressions for the three rates of division derived above for the hypothesis of probabilisitic binomial distribution of cell differentiation fulfill the requirement of $pp+pd+dd=1$.

$$(\frac{\Delta P+\frac{1}{2}\Delta D}{\Delta D+\Delta P})^2+\frac{\Delta D(2\Delta P+\Delta D)}{2(\Delta P+\Delta D)^2}+(\frac{\Delta D}{2(\Delta D+\Delta P)})^2 = 1 \tag{94}$$

extracting the common denominator, we obtain,

$$4(\Delta P+\frac{1}{2}\Delta D)^2+2\Delta D(2\Delta P+\Delta D)+\Delta D^2 = 4(\Delta D+\Delta P)^2 \tag{95}$$

rearranging terms:

$$4\Delta P^2+4(\frac{1}{2}\Delta D)^2+8\Delta P\frac{1}{2}\Delta D+4\Delta D\Delta P+2\Delta D^2+\Delta D^2 = 4(\Delta D+\Delta P)^2 \tag{96}$$

which can be simplified as

$$4\Delta P^2+4\Delta D^2+8\Delta P\Delta D = 4(\Delta D+\Delta P)^2 \tag{97}$$

Derivation of rates of division dependence on $pp - dd$

To obtain eqs. 8-10 in the main text, we start with the equation derived using the binomial approach (eq. 87) and the identity $pp + pd + dd = 1$.

$$(pp - dd)^2 = 1 - 2pd = 1 - 2(1 - pp - dd) = 2pp + 2dd - 1 = 2(pp - dd) + 4dd - 1 \tag{98}$$

where the second term has been rewritten as function of $pp - dd$. We can solve the second degree equation as:

$$pp - dd = \frac{2 \pm \sqrt{2^2 - 4(1 - 4dd)}}{2} = 1 \pm 2\sqrt{dd} \tag{99}$$

which gives us eq. 11 in the main text. Additionally, to obtain eq. 9 in the main text, we use the definition $pp + pd + dd = 1$, and substitute eqs. 9 and 11 in the main text.

$$pp = 1 - pd - dd = 1 - \frac{1 - (pp - dd)^2}{2} - \left(\frac{pp - dd - 1}{2}\right)^2 = ...$$
$$... = \frac{1}{2}\left(1 + (pp - dd)^2 - \frac{(pp - dd)^2 + 1 - 2(pp - dd)}{2}\right) \tag{100}$$

that after rearranging terms becomes eq. 11 in the main text.

References

[1] Saade, M. *et al.* Sonic Hedgehog Signaling Switches the Mode of Division in the Developing Nervous System. *Cell reports* **4**, 492–503 (2013).

[2] Le Dréau, G., Saade, M., Gutiérrez-Vallejo, I. & Martí, E. The strength of SMAD1/5 activity determines the mode of stem cell division in the developing spinal cord. *Journal of Cell Biology* **204**, 591–605 (2014).

[3] Kicheva, A. *et al.* Coordination of progenitor specification and growth in mouse and chick spinal cord. *Science* **1254927** (2014).

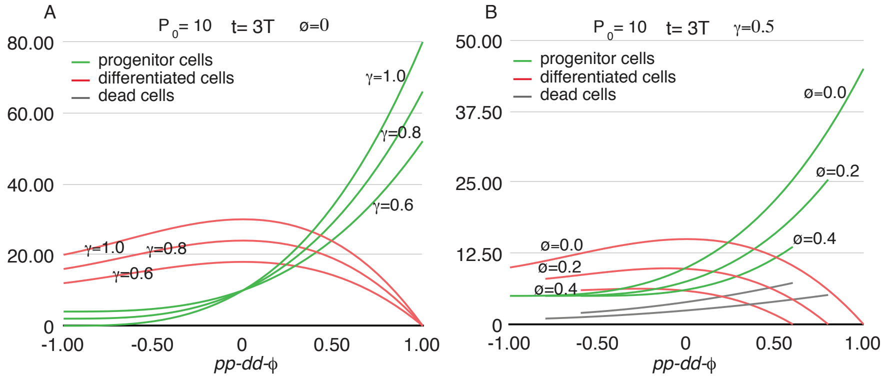

Figure 1: *
Supplementary Figure 1: Dependence of numbers of cells at a given time as a function of $pp - dd - \emptyset$ at different times for (a) different values of γ, (b) different values of $\emptyset$. (note that the system is only defined for $abs(pp - dd) > 1 - \emptyset$)

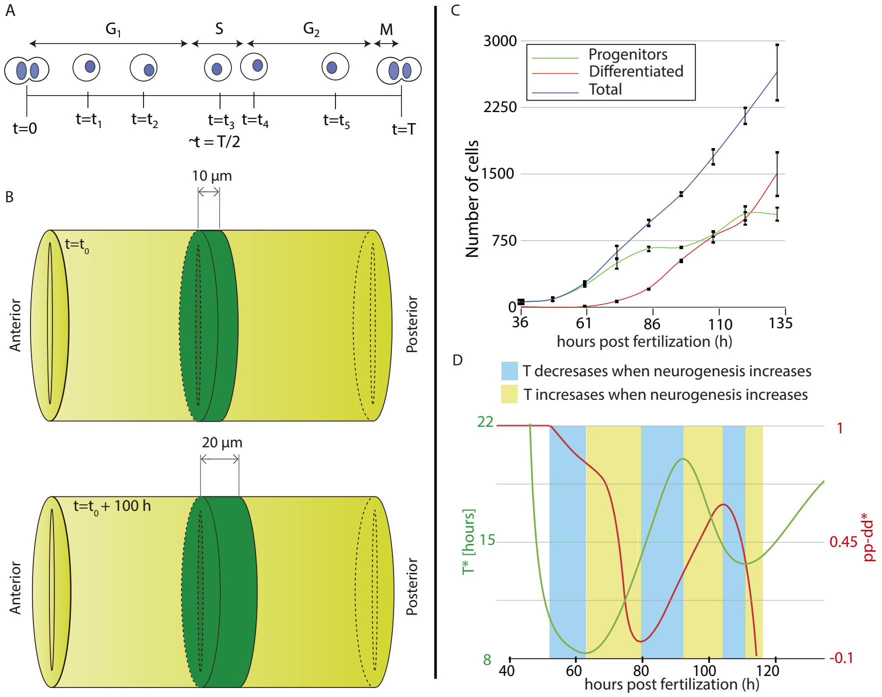

Figure 2: *

Supplementary Figure 2:(a) Scheme of time rescaling to compare experimental and theoretical data. For a single measurement in a single cell, the time passed since its generation is not known. When we perform multiple measurements in a population, some cells in a population will be in G1 phase and closer to its birth time in mitosis. Other cells will be older in time, going through S or G2 phases and approaching another mitosis, which is a full cell cycle time after its generation. When we ask this question simultaneoulsy to many cells in a asynchronous population, the average time passed since birth is 1/2 of the average cell cycle T (see Methods section for a detailed explanation) (b) Illustration of anteroposterior spinal cord growth and impact on quantification of cell cycle. Quantification is based on confocal sections of 10 μm (see Supplementary text for a detail explanation). (c) Cell numbers calculated taking into account the amount of cells generated in a given section that are lost to contribute to the anteroposterior growth of the spinal cord (100% extra number of cells after 100 hours of development, following data from (3)). (d) Superimposed curves of the effective T and pp-dd overtime.Yellow corresponds to regions where cell cycle length increases when differentiation increases. Blue corresponds do regions where the cell cycle length decreases when differentiation increases.

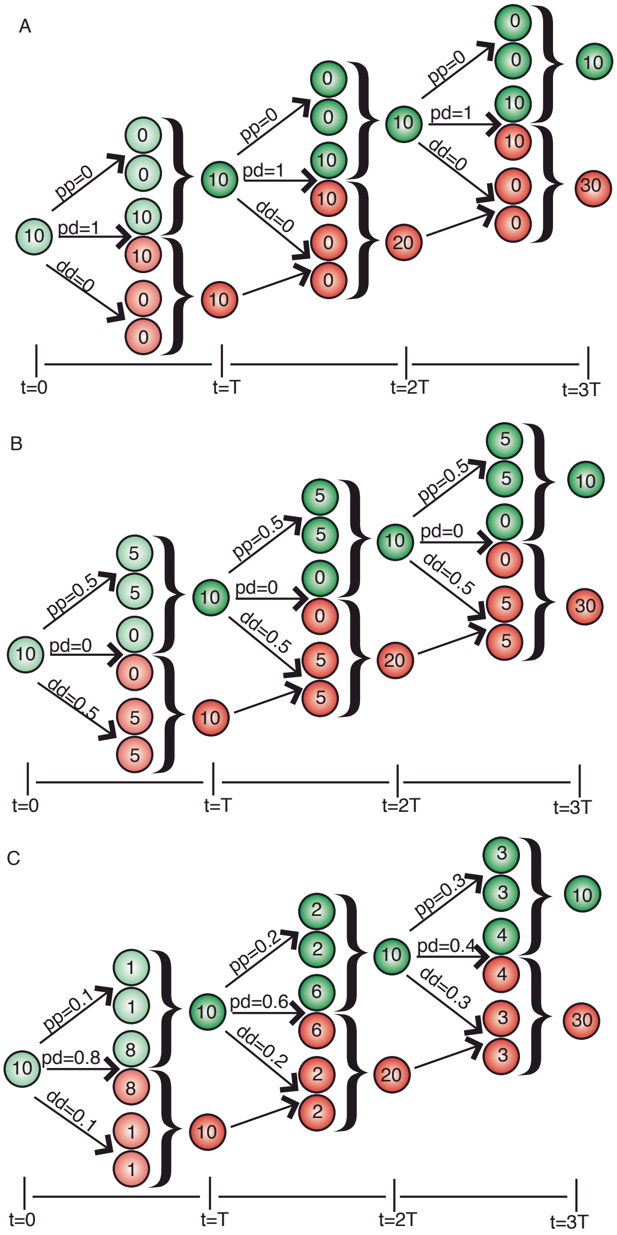

Figure 3: *

Supplementary Figure 3: Redundancy in the mode of division between *pp*, *pd* and *dd*. Three conditions where the same value of $pp - dd = 0$ is maintained but varying the balance between symmetric and asymmetric divisions produce the same final numbers of progenitors and differentiated cells after three rounds of divisions with: (a) all divisions being asymmetric, (b) all divisions being symmetric, and (c) changing the mode of division overtime but maintaining the same balance of $pp - dd = 0$ in all situations.

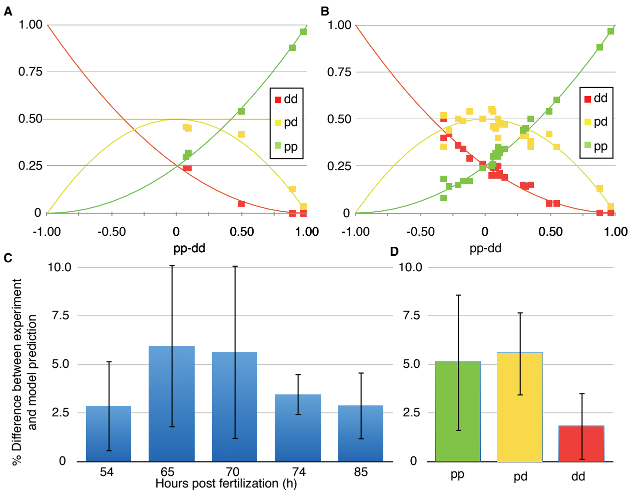

Figure 4: *
Supplementary Figure 4: Statistical comparison between experimental and theoretical data. (a) Plot of the experimental data (dots) in (2) on top of the prediction by the binomial hypothesis (lines). Average deviation, taking into account all five data points is 1.1% for *pp*, 2.8% for *pd* and 1.3% for *dd*. (b) Plot of the experimental data (dots) in all conditions (wt and mutants) in (2) for the whole tube and in (1) for motorneurons on top of the prediction by the binomial hypothesis (lines). The good agreement between experimental data and the prediction based on a binomial distribution suggests a scenario of independent probability of differentiation of daughter cells. (c-d) Difference between experiment (Fig 3D) and model prediction (Fig 3E) in percentage grouped by (c) developmental stage and (d) mode of division. Average difference is 5.1% for *pp*, 5.5% for *pd* and 1.8% for *dd*.

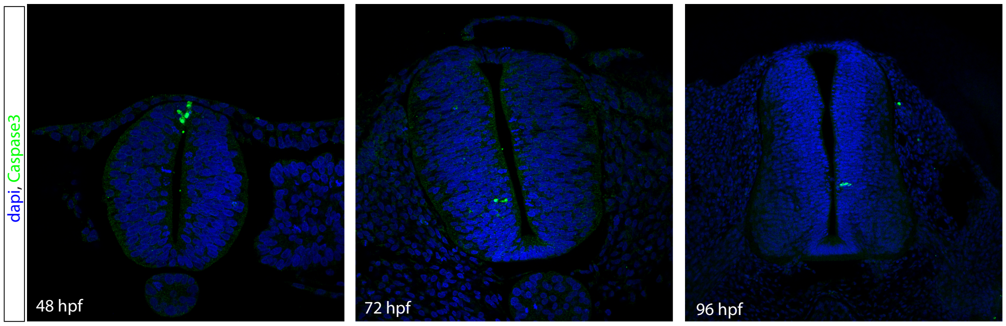

Figure 5: *
Supplementary Figure 5: Caspase3 (green) staining for representative spinal cord sections at 48, 72 and 96 HPF. Blue corresponds to dapi nuclear staining.

Figure 6: *
Supplementary Movie 1: Dynamics of the phenomenological simulation of the developing spinal cord ventricular zone, in conditions of growth in the progenitor population (pp-dd=0.5). Simulations have been performed as explained in detain the Methods section, maintaining all parameters constant. Initial conditions correspond to progenitor 30 cells distributed uniformly at different phases of the cell cycle. Apical domain where mitosis takes place is located on the left and basal domain on the right. Cells undergoing pp, pd and dd division as labeled as green, yellow and red, correspondingly. Differentiated cells are labeled as purple.

Figure 7: *

Supplementary Movie 2: Dynamics of the phenomenological simulation of the developing spinal cord ventricular zone, in conditions of homeostasis in the progenitor population (pp-dd=0).Simulations have been performed as explained in detain the Methods section, maintaining all parameters constant. Initial conditions correspond to progenitor 30 cells distributed uniformly at different phases of the cell cycle. Apical domain where mitosis takes place is located on the left and basal domain on the right. Cells undergoing pp, pd and dd division as labeled as green, yellow and red, correspondingly. Differentiated cells are labeled as purple.
